# Physical activity enhances theta-periodicity of visual attentional allocation

**DOI:** 10.1016/j.isci.2026.116240

**Published:** 2026-06-08

**Authors:** Xinyun Che, Christoph Reichert, Robert T. Knight, Stefan Dürschmid

**Affiliations:** 1 Leibniz Institute for Neurobiology, Brenneckestr. 6, 39118 Magdeburg, Germany; 2 Departments of Neuroscience and Psychology, University of California, Berkeley, 245 Warren Hall, Berkeley CA 94720, USA; 3 Helen Wills Neuroscience Institute, University of California, Berkeley, Berkeley CA 94720, USA; 4 Department of Psychiatry and Psychotherapy, Otto-von-Guericke University Magdeburg, Leipziger Str. 44, 39120 Magdeburg, Germany

**Keywords:** Physical activity, Cellular neuroscience, Sensory neuroscience

## Abstract

The traditional notion of a sustained visual spotlight of attention has been challenged by behavioral and neural evidence showing that attention fluctuates rhythmically in the theta band (3–8 Hz). In rodents, locomotion drives hippocampal theta oscillations that support cue sampling during navigation, enhancing sensory encoding and decision-making. This raises the question of whether physical activity (PA) similarly modulates theta-based attentional sampling in humans. Using magnetoencephalography (MEG), we found that discrimination accuracy (DA) increased following PA, accompanied by stronger theta-band modulation of both DA and reaction times (RTs) in trial-by-trial spectral analyses. Critically, PA elevated frontal theta power after cue presentation and shifted the coupling of visual high-frequency activity (HFA; 80–150 Hz) toward an encoding-sensitive theta phase. Moreover, target-evoked HFA exhibited stronger theta-range fluctuations after PA. Together, these findings demonstrate that short bouts of movement can tune the brain’s intrinsic theta-band sampling mechanism, thereby linking PA to enhanced visual attention.

## Highlights

• Physical activity enhances the theta-rhythmic modulation of sustained visual attention
• Walking speed predicts reaction times after PA, reflecting a state effect
• PA elevates frontal theta and shifts occipital HFA toward the encoding-sensitive phase
• Target-evoked HFA exhibits stronger theta fluctuations and lower noise after PA

## Graphical abstract

**Figure:**
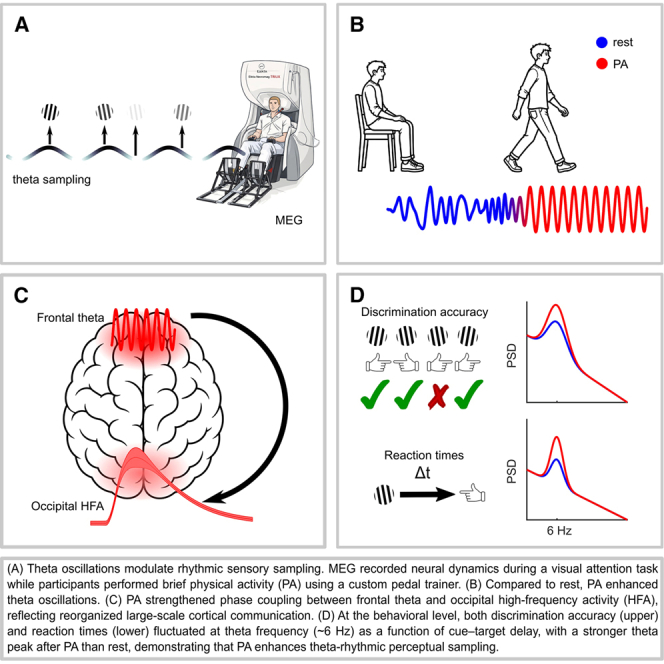

## Introduction

Visual attention has traditionally been conceptualized as a continuous process that enhances perceptual sensitivity at behaviorally relevant locations, the so-called spotlight of attention model. However, recent evidence suggests that sustained attention operates in an additional, discrete, rhythmic manner within the theta frequency band (3–8 Hz).^1^^,^^2^^,^^3^^,^^4^ Accordingly, the brain samples sensory information in cycles, resulting in fluctuations in performance that align with the phase of ongoing neural oscillations.^5^ This rhythmic sampling framework has been supported by findings from spatial cueing and visual search tasks, where perceptual sensitivity waxes and wanes in a theta-rhythmic manner.^1^^,^^3^^,^^6^^,^^7^^,^^8^

Theta oscillations are evolutionarily conserved across species and have been extensively characterized in rodents, where hippocampal theta is tightly linked to active exploration and voluntary locomotion.^9^^,^^10^^,^^11^^,^^12^ During movement, theta power increases and scales with running speed,^13^^,^^14^ suggesting a role in optimizing the processing of spatially relevant sensory input.^15^ In humans, it is unknown whether physical activity (PA) modulates perceptual theta-rhythmicity. An open question is whether brief bouts of PA—such as short walking periods—enhance the periodicity of visual processing by promoting rhythmic theta sampling in humans.

Here, we tested the hypothesis that a brief period of walking, referred to as PA, enhances a rhythmic mode of visual perception in humans. We asked whether movement shortly before experimental blocks—each consisting of consecutive trials lasting approximately 7 min—enhances perceptual rhythmicity in the theta band. This would provide evidence that theta oscillations in humans reflect a functional brain state that temporally structures behavioral sampling.

## Result

**Figure 1:**
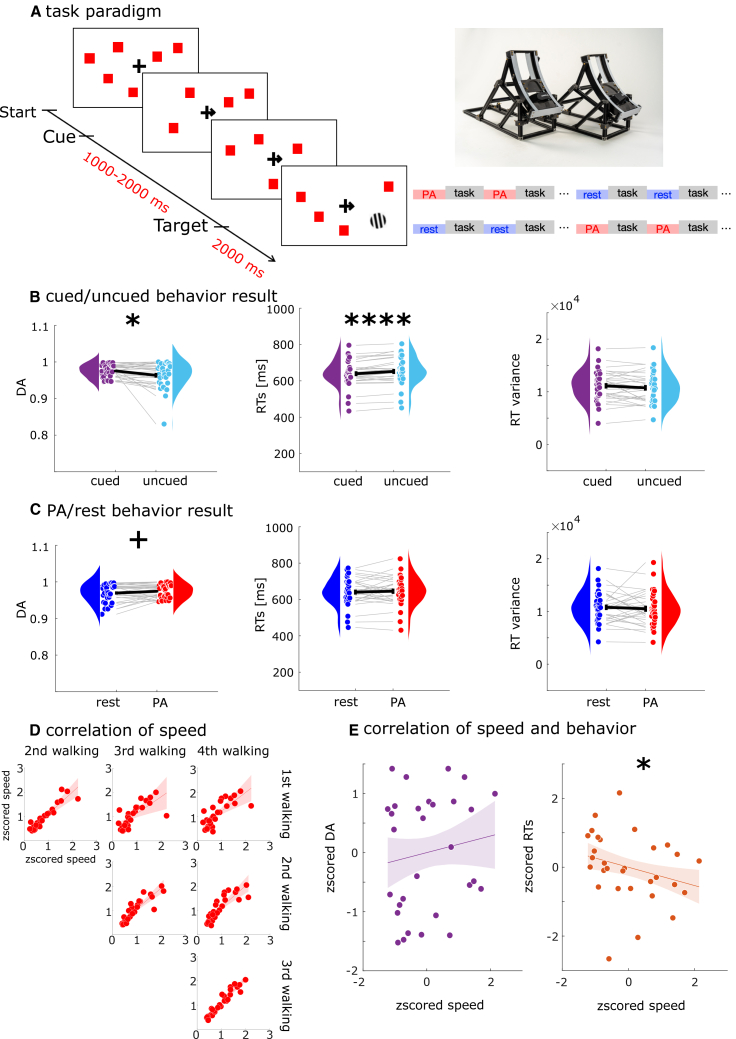
Task paradigm and behavior results (A) Depiction of the starry night paradigm (left), which was carried out after rest or walking on the MEG-compatible pedal trainer (top right). The experiment consisted of two sessions. Before each of the four main experimental blocks, participants either rested or walked using a pedal trainer, followed by performing the discrimination task. The order of rest and PA was counterbalanced (bottom right). (B) DA (left), RTs (middle), and RT variance (right) to targets at cued and uncued sides. These results indicate that participants consistently performed better at the cued trials, while maintaining high performance at both cued and uncued sides. Error bars indicate the standard error of the mean. (C) The same for trials after rest and PA. (D) Correlation of individual participants’ walking speeds across blocks. (E) The correlation between individual walking speed and DA after PA. Each dot represents an individual result, and the line indicates the linear regression of the data (∗*p* < 0.05, +*p* = 0.078, ∗∗∗∗*p* < 0.0001).

Thirty-one participants performed the Starry Night paradigm.^6^ In each trial (see Figure 1A), participants were presented with a spatial cue indicating the attended side (left or right), followed by a target grating after a variable cue-to-target delay (1000–2000 m s). Participants were informed that the target would appear on the cued side on most trials (70%). We compared behavioral performance between cued and uncued target locations to assess whether participants attended both hemifields simultaneously while preferentially allocating attention to the cued side. Gender differences were assessed (see Table S1), and no significant effects were found that affected the main conclusions. They were instructed to report the orientation of the target grating (leftward or rightward tilt) as quickly and accurately as possible using their right hand (index or middle finger, respectively).

### Comparison of cued and uncued target sides

We first compared behavioral performance between cued and uncued trials. For this analysis, discrimination accuracy (DA) was averaged across PA and rest sessions, as movement state was not treated as an independent factor at this stage. This approach allowed us to isolate the main effect of cueing while maximizing the number of trials contributing to each estimate. Participants had higher DA for cued compared to uncued targets (see Figure 1B, left; cued: *M* = 0.98, *SD* = 0.02; uncued: *M* = 0.96, *SD* = 0.04; *t*_(30)_ = 2.22, *p* = 0.034, BF_10_ = 1.61, η^2^ = 0.14, 95% CI [9.57 × 10^−3^, 0.02]). In addition, reaction times (RTs) were faster for cued targets (see Figure 1B*, middle*; cued: *M* = 630.45 m s, *SD* = 75.210; uncued: *M* = 651.74 m s, *SD* = 77.83; *t*_(30)_ = 4.95, *p* < 0.0001, BF_10_ = 848.8, η^2^ = 0.45, 95% CI [-17.36, −7.22]). However, RT variance did not differ between cued and uncued trials (see Figure 1B, right; cued: *M* = 1.11 × 10^4^, *SD* = 2.89 × 10^3^; uncued: *M* = 1.07 × 10^4^, *SD* = 2.95 × 10^3^; *t*_(30)_ = 1.53, *p* = 0.14, BF_10_ = 0.55, η^2^ = 0.07, 95% CI [-126.43, 879.72]). These results indicate that participants performed better in the cued trials, while maintaining high performance at both cued and uncued sides—a necessary condition for revealing rhythmic sampling effects.

### Comparison of PA and rest

We then compared overall performance between PA and rest sessions. Since both cued and uncued sides showed near-ceiling accuracy (>96%), we analyzed whether performance differed between PA and rest across both cued and uncued trials. We found a trend toward higher DA following PA (see Figure 1C, left; *PA*: *M* = 0.97, *SD* = 0.02; *rest*: *M* = 0.97, *SD* = 0.02; *t*_(30)_ = 1.84, *p* = 0.076, BF_10_ = 1.64, η^2^ = 0.1, 95% CI [-9.8 × 10^−3^, 5.19 × 10^−4^]), noteworthy given the high baseline DA in the rest session. However, there was no significant difference in mean RTs between PA and rest (see Figure 1C, *middle*; *PA*: *M* = 645.45 m s, *SD* = 80.25; *rest*: *M* = 640.55 m s, *SD* = 76.6; *t*_(30)_ = −0.62, *p* = 0.542, BF_10_ = 0.23, η^2^ = 0.01, 95% CI [-21.15, 11.34]), nor in RT variance (see Figure 1C, right; *PA*: *M* = 1.08 × 10^4^, *SD* = 2.9 × 10^3^; *rest*: *M* = 1.05 × 10^4^, *SD* = 3.21 × 10^3^; *t*_(30)_ = 0.57, *p* = 0.57, BF_10_ = 0.22, η^2^ = 0.01, 95% CI [-693.07, 1.24×10^3^]). Note that the absence of an overall reduction of RTs does not preclude the possibility of changes in RTs rhythmicity, a hypothesis we address in the following section.

### Individual speed

We next investigated whether inter-individual variability in walking speed accounts for differences in behavioral performance as indexed by DA and RTs. We tested whether walking speed correlated with performance during a rest session without concurrent PA. The correlation analysis between walking speed and behavioral performance during the rest session was conducted to assess whether walking speed reflects a stable, trait-like individual characteristic rather than a transient state effect. If walking speed correlated with performance during rest, this would suggest that individual differences in baseline walking speed are associated with general cognitive performance, independent of concurrent PA. By contrast, a correlation emerging specifically during the PA session would be more consistent with a state-dependent effect, in which physical exertion transiently modulates cognitive performance. Thus, the rest-session analysis serves as a critical control, allowing us to dissociate trait-related individual differences from PA-induced state-dependent modulation. This distinction strengthens the interpretation that the observed PA-related effects reflect transient cognitive modulation rather than stable behavioral traits. Instantaneous walking speed was recorded using a light-barrier sensor attached to the pedal trainer. On average, participants maintained a moderate speed of 0.95 steps per second throughout the experiment. First, we assessed the consistency of walking speed across the four PA blocks. For each participant, average speed was calculated separately for each block (see methods). Pairwise correlations across all blocks revealed high intra-individual consistency (all Spearman’s ρ > 0.7, all *p* values <0.0001; see Figure 1D). Given this consistency, we averaged walking speed across blocks for each participant. We did not find a significant correlation with RTs (see Figure S2, right; r_(29)_ = −0.15, *p* = 0.421) or DA (see Figure S2, left; r_(29)_ = 0.03, *p* = 0.884) when we correlated average speed with performance in the rest session. In contrast, while walking speed was not correlated to DA (r_(29)_ = 0.14, *p* = 0.475; see Figure 1E, left) in the PA session, individual speed predicted RTs, with faster individual speed associated with shorter individual RTs (Spearman’s r_(29)_ = −0.39, *p* = 0.035; see Figure 1E, right). The observation that a significant correlation emerged only during the PA session suggests that this relationship is driven by a PA-induced state effect rather than a stable behavioral trait.

### Theta rhythmic modulations in behavioral performance

**Figure 2:**
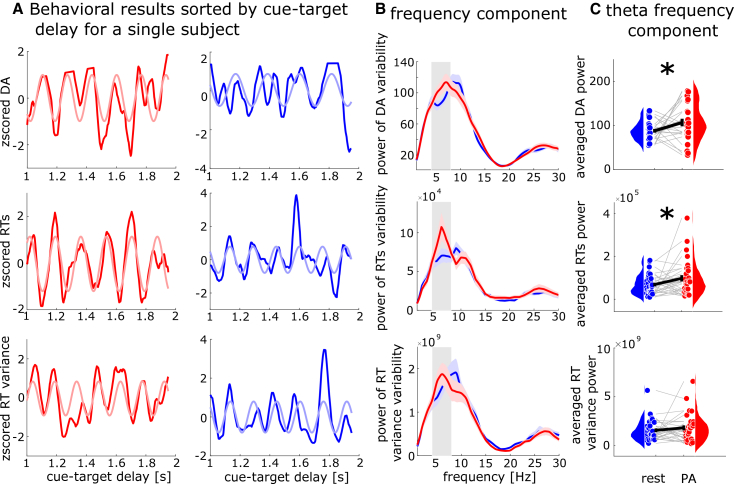
The rhythmic fluctuation of behavioral performance as a function of cue-to-target delay (A) Single-subject examples of behavioral performance rhythm as a function of the cue-to-target delay. The light-colored line indicates the unconstrained cosine fit to the data. Blue indicates rest session, and red indicates PA session. (B) Group-level 1/f-corrected power spectra of the behavioral time courses. The gray shading highlights the 5–7 Hz frequency range. (C) Comparison of PSD values between rest and PA averaged between 5 and 7 Hz. Error bars indicate the standard error of the mean (∗*p* < 0.05).

We sorted trials according to cue-to-target delay, grouped them into 50 m s bins with a 2 m s sliding step, and extracted mean DA, mean RTs, and RT variance in each bin to examine whether performance exhibited oscillatory. This resulted in a time series of 476 values for each participant (see methods). DA, RTs, and RT variance exhibited regular phases of waxing and waning over time in individuals (see Figure 2A). To quantify rhythmicity, we applied a fast Fourier transform (FFT) to convert the detrended time series into power spectral density (PSD; see Figure 2B). We averaged PSD between 5 and 7 Hz (see Figure 2C) and compared the resulting values between PA and rest. We found a stronger modulation of RTs after PA (t_(30)_ = 1.83, *p* = 0.039, BF_10_ = 1.62, η^2^ = 0.1, 95% CI [-Inf, −2×10^3^]), indicating stronger theta-band modulation of behavioral performance after PA. No significant PSD difference was observed for RT variance (t_(30)_ = 0.97, *p* = 0.169, BF_10_ = 0.49, η^2^ = 0.03, 95% CI [-Inf, 1.88×10^8^]). Some participants showed perfect DA (100% accuracy), producing ceiling effects that obscured rhythmic modulations due to the absence of performance variability. To address this, we computed the variance of DA across the cue-to-target interval for each participant. Six participants with the lowest variance (<20^th^ percentile)—reflecting consistently high performance—were excluded from further rhythmicity analyses on DA. PSD was higher following PA compared to rest for DA (t_(24)_ = 1.81, *p* = 0.042, BF_10_ = 1.65, η^2^ = 0.12, 95% CI [-Inf, −0.98]). Note that this exclusion criterion applied only to the DA rhythmicity analysis due to the absolute upper limit of 100%. In supplementary analyses of rhythmicity over time (see Figure S3), we estimated PSD in successive intervals, quantified temporal slopes, and found that DA rhythmicity decreased during rest but not after PA, with conditions differing significantly. Notably, differences in all three behavioral measures (DA, RTs, RTvar) between PA and rest overlapped, with the most pronounced difference observed at 6 Hz (see Figure S4).

### Target response

**Figure 3:**
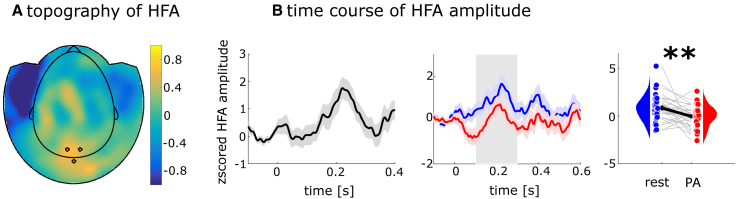
HFA in the occipital region (A) Topography of the HFA amplitude averaged across all trials and all participants within the 0–400 m s window following target onset. (B) (left) The time course of HFA amplitude around target onset, averaged across all trials. (middle): The time course of HFA amplitude for PA and rest sessions. (right): scatterplot of individual HFA amplitude averaged from 100 to 300 m s. The red color represents signals from PA sessions, while the blue color represents signals from rest sessions, and error bars indicate the standard error of the mean (∗∗*p* < 0.01).

We focused on the high-frequency activity (HFA) (80-150 Hz), which is linked to selective attention in humans^16^ and correlates with local population neural firing rate and cortical excitability.^17^^,^^18^ Importantly, occipital HFA integrates both bottom-up visual input and top-down feedback from frontal control regions,^16^^,^^19^^,^^20^^,^^21^^,^^22^ and has been shown to be rhythmically modulated by theta-band oscillations during attention.^3^ We therefore used occipital HFA as a sensory-level index of attentional gain, allowing us to assess how PA-related modulation of theta dynamics impacts visual processing. We observed an HFA increase shortly after target onset in the occipital region (see Figure 3A), where a prominent peak was observed in a cluster of three magnetoencephalography (MEG) sensors covering the occipital region of interest between 100 and 300 m s. When we compared HFA after PA and rest, we found a smaller amplitude after PA (see Figure 3B; *PA*: 8.64 × 10^−17^ ft; *rest*: 4.46 × 10^−16^ ft, t_(30)_ = 3.05, *p* = 0.005, BF_10_ = 8.38, η^2^ = 0.24, 95% CI [0.29, 1.49]). We reasoned that the observed reduction in amplitude is due to an overall decrease in target-unrelated noise in the HFA following PA. We hypothesized that reduced target-unrelated noise would increase the similarity of HFA amplitude across participants. During the rest sessions, pairwise inter-participant correlations of the HFA time courses were not significantly different from zero (see Figure S5, right; mean *r* = −0.007, *t*_(464)_ = 0.43, *p* = 0.67, BF_10_ = 0.06, η^2^ = 3.91 × 10^−4^, 95% CI [-0.04, 0.03]) indicating that HFA time courses are not correlated across subjects. However, we found a significant increase in pairwise inter-participant correlations, indicating higher similarity across participants following PA (mean *r* = 0.072, *t*_(464)_ = 4.57, *p* < 0.00001, BF_10_ = 1325.6, η^2^ = 0.04, 95% CI [-0.12, 0.1]), indicating increased cross-subject HFA similarity. Furthermore, correlation among participants was higher in the PA session compared to rest (*t*_(464)_ = 3.63, *p* < 0.001, BF_10_ = 33.31, η^2^ = 0.03, 95% CI [-0.12, −0.04]), indicating a PA-induced reduction in stimulus-unspecific, individual variability.

### Theta modulation after cue onset

We tested whether the theta-rhythmic modulation of behavior reflects PA-induced changes in preparatory control states indexed by post-cue theta activity. Theta-band activity has been linked to cue-driven attentional sampling mechanisms that operate prior to target detection.^23^ Accordingly, theta activity was analyzed during the post-cue, pre-target interval, which reflects a sustained preparatory attentional state in which theta-based sampling is active before stimulus presentation. Activity in this interval captures neural dynamics that shape subsequent sensory processing^24^ and contribute to behavioral variability at target onset. Focusing on this pre-target period also minimizes confounding influences from target-evoked eye movements, feedback-related processing, and response-related motor activity. Thus, the post-cue interval provides a selective measure of attentional rhythmicity and its modulation by PA, independent of sensory-evoked and motor-related neural responses.

**Figure 4:**
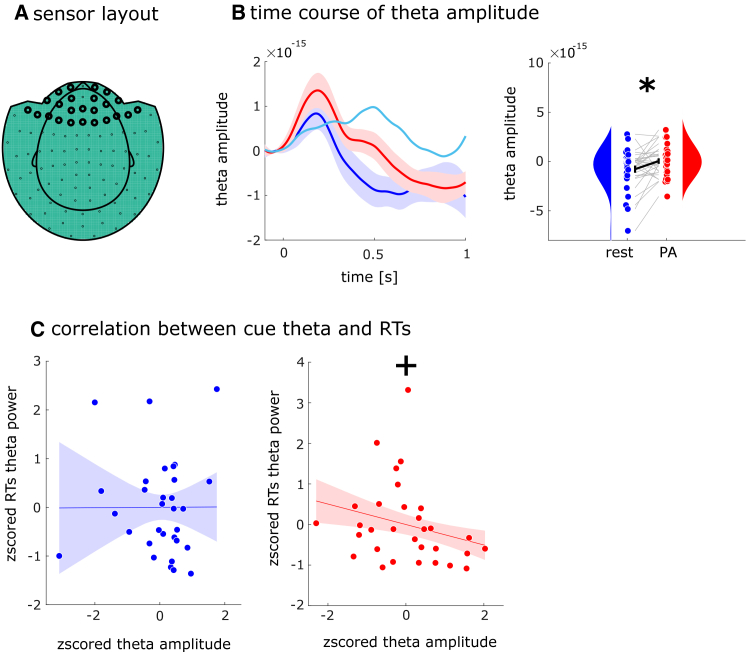
Frontal theta amplitude following cue onset (A) Sensor layout over the frontal cortex. (B) (Left) Grand-average theta amplitude time course aligned to cue onset. Red and blue lines indicate PA and rest sessions, respectively. The cyan line represents the difference in theta amplitude between PA and rest sessions. (Right): Individual mean theta amplitude averaged across the shaded time window. Error bars indicate the standard error of the mean (∗*p* < 0.05). (C) Correlation between theta amplitude after cue onset and RTs theta power from power spectral analysis (+*p* = 0.056).

We first tested whether PA modulated theta activity after cue onset. To avoid contamination from cue-evoked sensory responses and phase-reset effects, theta amplitude from 360 m s (two 6 Hz cycles) to 1 s (1^st^ target onset) was compared between rest and PA across four regions of interest (frontal, temporal, parietal, and occipital; see Figure 4; Figure S6). Theta amplitude in the frontal region was higher in the PA compared to the rest session (see Figure 4B; t_(30)_ = 2.74, *p* = 0.01, BF_10_ = 4.35, η^2^ = 0.2, 95% CI [-1.43 × 10^−15^, −2.08 × 10^−16^]). No significant effects were observed in the other regions (see Figure S6).

We examined whether individual differences in post-cue frontal theta, indexing preparatory control, accounted for variability in behavioral performance. The frontal cortex, a key node of the frontoparietal attention network, has been shown to influence theta-band rhythmic attentional sampling.^6^ For each session (rest and PA), we separately correlated individual frontal theta amplitude (averaged 360–1000 m s after cue onset) with theta power in RTs derived from PSD analysis. No significant relationship was observed in the rest session (see Figure 4C, left; r = −0.05, *p* = 0.787). However, the PA session showed a trend toward a negative correlation (see Figure 4C, right; r = −0.35, *p* = 0.056), suggesting that PA-modulated preparatory attentional states may be linked to later behavioral theta sampling.

We next assessed phase-amplitude cross-frequency coupling between frontal theta phase and simultaneous occipital HFA amplitude during the cue-to-target interval. Theta-HFA coupling strength, quantified by the circular concentration parameter *κ*, did not differ between sessions (*rest*: k = 0.04; *PA*: k = 0.23; *p* = 0.48). However, the preferred theta phase of maximal HFA differed significantly (*rest*: *μ* = −1.8 rad; *PA*: *μ* = 1.9 rad; *p* = 0.0014; see Figure S7), with HFA aligned to the ascending theta phase during rest and to the descending phase during PA.

### Theta rhythmic modulations of HFA

**Figure 5:**
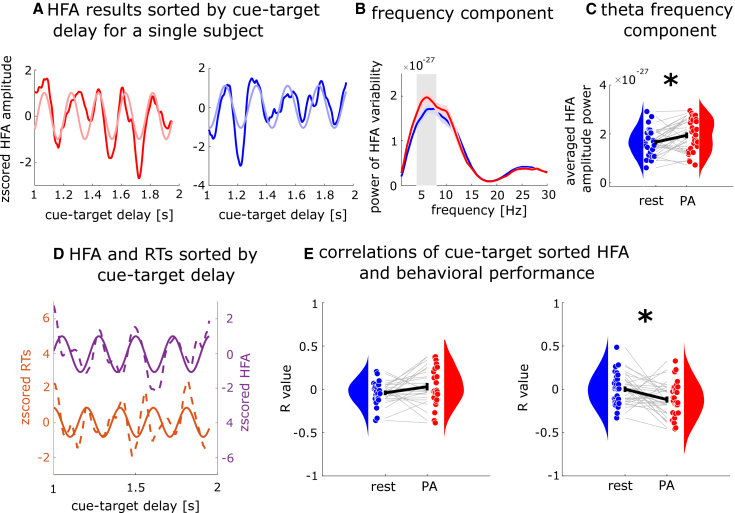
Cue-target interval-sorted HFA amplitude (A) An example of HFA amplitude sorted by cue-to-target delay for PA sessions (left) and rest sessions (right), with the light-colored line indicating the fitted cosine wave. (B) Group-level 1/f-corrected power spectra of the HFA amplitude sorted by cue-to-target delay. The gray shading highlights the 4–8 Hz frequency range. (C) Scatterplot of individual PSD averaged within the center of the theta band (∗*p* < 0.05). (D) RTs and HFA sorted by cue-target delay from a single subject. Orange and purple lines represent RTs and HFA, respectively. The solid line indicates the cosine fit, while the dashed line shows the actual data. (E) (Left) correlation with DA. (Right): correlation with RTs. Red and blue indicate PA and rest sessions, respectively. Error bars indicate the standard error of the mean (∗*p* < 0.05).

Next, we examined whether HFA target-response in the occipital region demonstrates stable or oscillatory modulation patterns aligned with cue-to-target intervals, analogous to behavioral measures. We sorted the HFA (averaged between 100 and 300 m s) according to cue-to-target delay as outlined for the behavioral performance (see Figure 5A). The resulting HFA cycles were then transformed into PSD (see Figure 5B), revealing prominent theta-band fluctuations peaking at ∼6 Hz (see Figure 5B), matching behavioral oscillation patterns (see Figure 2B). We observed a higher amplitude within theta-band power (5–7 Hz) in the PA session (see Figure 5C, t_(30)_ = 2.60, *p* = 0.015, BF_10_ = 3.2, η^2^ = 0.18, 95% CI [-4.89 × 10^−28^, −5.79 × 10^−29^]). Most importantly, both HFA and behavioral performance show the strongest PA-rest difference in a narrow band peaking around 6 Hz (see Figure S4).

### HFA fluctuations track behavioral oscillations under PA

We then examined whether fluctuations in HFA covary with rhythmic variations in behavior. For each participant, we computed the correlation between the phase of HFA cycles and behavioral oscillations (RTs and DA; see Figure 5D), and then compared the resulting correlation coefficients across the rest and PA sessions. No effect was found between PA and rest in the correlations between HFA and DA (see Figure 5E, left; t_(30)_ = 1.61, *p* = 0.121, BF_10_ = 0.65, η^2^ = 0.1, 95% CI [-0.16, 0.02]). However, HFA fluctuations were negatively correlated with RTs fluctuations in the PA session (see Figure 5E, right; mean r = −0.118, t_(30)_ = 3.34, *p* = 0.002, BF_10_ = 16.18, η^2^ = 0.27, 95% CI [-0.19, −0.05]), indicating that increased HFA coincided with faster responses. In contrast, no such relationship emerged during rest (mean r = 0.001, t_(30)_ = 0.04, *p* = 0.967, BF_10_ = 0.19, η^2^ = 5.65 × 10^−5^, 95% CI [-0.06, 0.07]). A direct comparison confirmed that the HFA-RT coupling was significantly stronger in PA than in rest (t_(30)_ = 2.46, *p* = 0.02, BF_10_ = 2.49, η^2^ = 0.16, 95% CI [0.02, 0.22]). Together, these results indicate that PA selectively enhances a shared theta-band (∼6 Hz) rhythmic process linking neural (HFA) and behavioral oscillations, suggesting a dynamic coupling between cortical excitability and behavioral performance.

## Discussion

We investigated the impact of PA on perceptual cycling and found that PA modulated perceptual performance. PA before performing the task improved DA compared with the rest of the session. Participants exhibited consistent individual speed profiles, and individual speed during PA predicted subsequent behavioral performance—an association not observed after rest—suggesting a state-dependent effect of PA. PA increased the narrow-band theta modulation of behavioral performance. Furthermore, PA decreased HFA amplitude but increased theta modulation of HFA to targets. DA and RTs showed stronger theta-band modulation after PA, and the largest differences between PA and rest occurred at 6 Hz. At the neural level, we observed enhanced theta activity over the frontal cortex contributing to behavioral theta modulation in the PA session. Furthermore, we found robust stimulus-locked HFA, which were reduced in amplitude but similar across participants after PA compared with rest. HFA showed a robust theta-band modulation after PA, and HFA amplitude predicted RTs differences only in the PA session, with higher HFA theta modulation linked to faster responses after sorted by cue-target delay.

While rodent studies report improved perception during PA, human findings are mixed. Benjamin et al. (2018) found higher contrast thresholds during treadmill walking than at rest.^25^ This difference may reflect methodological factors, as vertical head motion during walking can shift the visual field and affect stimulus alignment.^26^ Recent studies using spatial attention tasks,^27^ SSVEP,^28^ and alpha modulation^29^ report stronger neural responses during walking or light exercise, but no behavioral improvements, and in some cases, performance declines.^28^^,^^29^^,^^30^ These effects may reflect dual-task demands or head movement relative to visual stimulation.^26^ In sum, the inconsistent findings in human research may reflect the combined effects of movement-related visual field shifts and dual-task interference. We minimized visual field shifts by restricting head movement in the MEG environment. Moreover, in our study, the task was performed shortly after PA rather than during movement. By separating the movement phase from the cognitive task, we show that PA improves visual perception.

We found that perceptual cycling in the theta range increased following PA, and that movement speed predicted behavioral performance in the PA session. This pattern supports a movement-induced state change, driven by theta oscillations persisting after the cessation of movement. In humans, theta activity increases during movement^31^^,^^32^^,^^33^^,^^34^ and typically returns to baseline after locomotion,^31^^,^^35^^,^^36^ though some persistence has been reported.^35^ Similarly, in rodents, hippocampal theta is closely linked to locomotion and often declines rapidly after movement stops,^37^^,^^38^ yet several studies describe sustained or gradually decaying oscillatory activity. Our data demonstrate that cycling-related perception in the theta range persists even after movement has ceased. Together, these observations indicate that while theta is generally movement-locked, under certain behavioral or species-specific conditions, it can persist beyond locomotion, supporting the idea that our post-movement effects reflect an intrinsic cognitive rhythm rather than a movement artifact.

Here, we address rhythmic attention sampling the theta range (3–8 Hz)^1^^,^^3^^,^^39^^,^^40^^,^^41^^,^^42^^,^^43^^,^^44^^,^^45^ by testing how PA impacts theta rhythmicity in a paradigm previously shown to reveal theta-phase-dependent behavior, even at long stimulus-onset asynchronies (SOAs). Following PA, we observed a selective, narrowband increase in theta power (4–8 Hz)—without comparable changes in neighboring frequencies—which excludes broadband arousal and muscle artifacts as an explanation. Critically, behavioral performance varied systematically with the phase of this post-PA theta—an effect incompatible with purely serial or sustained-attention accounts. The persistence and phase specificity of this modulation, emerging after movement has ceased, demonstrates that the observed theta is not a movement artifact but an internally generated sampling rhythm. These findings provide causal evidence that attention is inherently rhythmic, with movement acting as a trigger that transiently amplifies theta-driven sampling.

Multiple intracranial and EEG studies provide converging evidence that human walking increases theta-band activity, but none of these investigations demonstrate that theta modulation translates into measurable changes in visual perception. Bohbot et al. (2017) reported robust 7–9 Hz hippocampal theta during real-world navigation,^36^ and Goyal et al. (2020) showed that high-theta (∼8 Hz) scales with walking speed,^46^ paralleling rodent type-I theta. Similarly, Aghajan et al. (2017) found higher theta occurrence during walking than at rest,^31^ and scalp EEG studies report widespread theta increases during whole-body movement.^33^^,^^47^ However, participants often moved only in virtual environments^46^ or without concurrent perceptual measures.^36^ Thus, although these studies confirm PA-related theta modulation, they do not demonstrate that this activity cyclically shapes visual perception. Here, we demonstrate that movement enhances the rhythmicity of perceptual cycling, specifically within the theta frequency range. Our findings suggest that, in humans, theta activity reflects a cue-sampling mechanism linked to active movement, supporting the view that perception and action are coordinated through rhythmic neural dynamics.

PA selectively increased frontal theta power during the cue-to-target interval, and individual frontal theta amplitude predicted the strength of behavioral theta modulation in RTs. In primates, including most human studies, theta-rhythmic fluctuations in perceptual sensitivity have been primarily attributed to a frontoparietal attention network—comprising the frontal eye fields, lateral intraparietal area, and mediodorsal pulvinar—which alternates between phases of heightened and reduced perceptual sensitivity.^1^^,^^2^ Frontal theta has been widely implicated in attentional control and the temporal organization of sensory processing, and is thought to reflect top-down coordination within the frontoparietal attention network.^23^^,^^24^ Neurophysiological studies in non-human primates^48^^,^^49^ and humans^2^^,^^3^^,^^50^ implicate theta oscillations in modulating neural excitability and dynamically reweighting functional connections. Our findings indicate that PA amplifies this preparatory theta activity, consistent with the interpretation that movement primes attentional control circuits and strengthens rhythmic sampling prior to stimulus onset.

HFA (80–150 Hz) is a broadband signal that correlates with local neuronal firing^51^ and reflects the magnitude and timing of sensory-evoked responses.^52^ In the visual system, it typically emerges before 100 m s, peaks before 200 m s, and decays gradually^53^^,^^54^^,^^55^^,^^56^—a profile also observed in MEG recordings.^57^ In our data, the HFA resembled the canonical profile but with a delayed onset, likely due to the small and brief stimuli used. We found a reduction in HFA amplitude during PA, a pattern more consistent with the locomotion-related suppression of V1 responses observed in marmosets^58^ than with the locomotion-induced enhancement in rodents, which has been attributed to disinhibition via specific inhibitory interneurons.^59^^,^^60^ This pattern suggests that MEG-HFA is not primarily driven by inhibitory neuron activity. This reduction may reflect altered feedback-related processing rather than a simple attentional gain effect. Moreover, HFA also exhibited higher similarity across subjects in the PA session, which could be explained by a reduction in neuronal noise—that is, stimulus-independent activity. In the visual cortex, interconnected cell populations tuned to specific features exhibit both stimulus-driven and noise correlations, the latter of which can impair^61^ or, under certain states, improve^62^ population coding. PA has been shown to modulate HFA^20^ and alter interneuronal correlation patterns across brain states. Furthermore, stimulus onset itself reduces neural variability, as reflected by a drop in the Fano factor, which measures the spike-count variance relative to its mean,^63^ and attention is known to reduce interneuronal correlations in early visual areas. Our findings suggest that PA, in addition to attention, can modulate HFA in a way that both suppresses HFA amplitude and reduces stimulus-independent variability, pointing toward a state-dependent reconfiguration of feedback processing in early visual cortex.

Beyond amplitude changes, occipital HFA (100–300 m s after target onset) exhibited enhanced theta-band modulation after PA and became tightly coupled to behavioral fluctuations in RTs. This pattern suggests that PA is associated with a selective linking of sensory cortical excitability and behavioral performance through a shared theta rhythm. In parallel, post-cue frontal theta activity was associated with subsequent theta-band power in RTs (as revealed by PSD analysis) following PA, indicating that PA-related increases in preparatory theta activity were behaviorally relevant already at the anticipatory stage. Together, these findings support a sequential, correlational framework in which PA-enhanced post-cue frontal theta activity may shape the subsequent sensory processing reflected in post-target occipital HFA, which in turn is associated with behavioral performance. These findings suggest that PA modulates the temporal organization of visual processing by aligning preparatory and sensory-related neural responses within a theta-rhythmic framework.

Our findings also demonstrate that PA reorganizes large-scale cortical coupling, reflected by a shift of occipital HFA coupling toward the descending phase of frontal theta oscillations relative to rest. This phase is linked to sensory encoding, whereas the ascending flank supports retrieval in hippocampal-cortical networks.^64^^,^^65^ Mechanistically, theta rhythms temporally segregate encoding vs. retrieval to reduce interference between externally driven input and internally generated memory traces. Consistent with this framework, locomotion biases neural dynamics toward an encoding state. Movement enhances theta stability/synchrony and sensorimotor integration,^66^ and can shift theta-gamma coupling toward earlier (descending) theta phases after movement onset.^67^ Human intracranial work likewise shows that active exploration modulates hippocampal theta and long-range coupling during movement.^46^^,^^68^ In line with this evidence, the post-movement shift of occipital HFA-frontal theta coupling to the descending phase in our data may reflect movement-induced optimization of feedforward sensory routing toward frontal control regions—a retuning of temporal coordination rather than local excitability, as overall HFA power was unchanged.

Together, these mechanistic considerations are supported by our key findings: brief PA enhances visual discrimination performance and selectively increases the theta-band modulation of both behavior and neural activity. Movement speed during PA predicted subsequent task performance, indicating a state-dependent effect not seen after rest. Crucially, PA amplified narrowband theta rhythmicity in perceptual cycling and target-evoked HFA, with theta phase predicting behavioral fluctuations only in the PA session. These results reconcile prior inconsistencies in human studies by showing that separating the movement phase from task execution eliminates visual field confounds and dual-task costs, revealing genuine post-movement benefits. Together, the data provide evidence that theta oscillations, triggered and sustained beyond movement, constitute an intrinsic sampling rhythm coordinating perception. Our study identifies a mechanism where movement reconfigures cortical states to optimize perceptual processing.

### Limitations of the study

Our study has several limitations that open avenues for future research. While MEG allowed us to capture the temporal dynamics of theta and HFA with high precision, its limited sensitivity to deep brain structures such as the hippocampus prevents definitive conclusions about the generators of the observed effects. Moreover, because we separated the movement and perceptual task phases to minimize dual-task confounds, our design may not fully capture how perception unfolds during naturalistic, continuous locomotion. The relatively brief and uniform PA intervention also constrains generalizability, leaving open the question of whether longer, repeated, or more ecologically valid forms of movement produce sustained benefits. Addressing these issues will require multimodal approaches, such as combining MEG with intracranial recordings, virtual reality paradigms, or longitudinal designs, to delineate how movement reshapes neural dynamics and perception across different timescales and contexts.

## Resource availability

### Lead contact

Further information and requests for resources and reagents should be directed to and will be fulfilled by the lead contact, Stefan Dürschmid (stefan.duerschmid@lin-magdeburg.de).

### Materials availability

This study did not generate new unique reagents.

### Data and code availability

• All data needed to evaluate the conclusions in the paper are available in the GitHub repository: https://github.com/FreyaChe/visual_attention_script. Raw and preprocessed MEG/EEG data are not publicly available due to participant privacy restrictions and ethics approval requirements. Requests for access should be directed to the lead contact and are subject to institutional data sharing agreements.
• All analysis code is available in the GitHub repository listed above https://github.com/FreyaChe/visual_attention_script.
• Any additional information required to reanalyze the data reported in this paper is available from the lead contact upon request.

## Acknowledgments

This work was supported by the Deutsche Forschungsgemeinschaft (DFG,German Research Foundation) – SFB 1436, Project A03 and PsychCircuits.

## Author contributions

X.C. and S.D. conceived and designed the experiment. X.C. collected the MEG data and performed data preprocessing and analysis. S.D. and C.R. supervised data analysis. X.C., C.R., R.T.K., and S.D. wrote the manuscript.

## Declaration of interests

The authors declare no competing interests.

## STAR★Methods

### Key resources table

**Table:** 

REAGENT or RESOURCE	SOURCE	IDENTIFIER
**Deposited data**		

Analyzed data	This paper	https://github.com/FreyaChe/visual_attention_script

**Software and algorithms**		

MATLAB	https://www.mathworks.com/products/matlab.html	Version R2016b
Psychophysics Toolbox	http://psychtoolbox.org	–
FieldTrip Toolbox	https://www.fieldtriptoolbox.org	–
Circular Statistics Toolbox	https://github.com/circstat/circstat-matlab	–
MATLAB Codes	This paper	https://github.com/FreyaChe/visual_attention_script

### Experimental model and study participant details

A total of 31 participants (19 female, 12 male; mean age = 26.54 years, SD = 3.32 years) took part in the visual perceptual experiment. All participants reported normal or corrected-to-normal vision and no history of neurological or psychiatric disorders. Furthermore, no physical impairments relevant to body movement were reported. All participants received detailed information about the study and provided written informed consent prior to participation. Recordings were conducted at the Department of Neurology, Otto-von-Guericke University Magdeburg, with approval from the local ethics committee (“Ethical Committee of the Otto-von-Guericke University Magdeburg”). All procedures complied with relevant ethical regulations for research involving human participants. Information on race, ethnicity, or ancestry was not collected as part of the study protocol. Therefore, potential influences of these factors on the results cannot be assessed.

### Method details

#### Paradigm and procedure

The stimulus presentation and experimental control were carried out using MATLAB R2009a (Mathworks, Natick, USA) and the Psychophysics Toolbox.^69^ The experiment consisted of four blocks per session, with each block containing 100 trials and lasting approximately 7 min. The screen background was set to white, with distractors displayed in red and the fixation cross, cue, and target displayed in black. Each trial began with a 500 m s baseline period during which all distractors were displayed on the screen. Participants were then cued to focus on either the left or right side by an arrow presented at the center of the screen, while distractors (n = 8–12) simultaneously began flashing at random intervals independently. The target grating appeared after a variable cue-to-target interval (1000–2000 m s), with a 70% probability of being presented on the cued side. The screen refresh rate was 120 Hz, corresponding to an 8.3 m s refresh interval, and the actual interval was calculated based on the photodiode signal from cue to target (see Figure 1A). Participants were instructed to report the direction of the grating as quickly and accurately as possible during a 2000 m s response window. They were instructed to use the right index and middle finger for left and right tilt orientations, respectively. The PA and rest sessions were conducted within the same recording session, with the sequence of PA and rest sessions randomly assigned to minimize bias. Participants began with either a full set of PA blocks or rest blocks where each block began with a 2-min phase of either rest or walking.

The pedal trainer was custom-built in-house for MEG recordings, with all materials carefully tested to ensure the absence of magnetic components. The device’s angle and height were adjustable to accommodate individual participant proportions, and its design allowed for independent forward and backward movement of both feet. A beam breaker at the pedal pad reflected a light signal to the response system (DataPixx) whenever participants moved their feet forward to the top position, enabling precise measurement of instantaneous speed. Participants were instructed to pedal at a moderate, self-paced rhythm resembling natural walking. This adjustment was implemented to minimize MEG artifacts, including ECG interference and changes in skin impedance due to sweating,^70^ which may also influence EEG electrode contact. Comparable moderate-intensity exercise paradigms, such as wheel running in rodents, have been shown to enhance hippocampal plasticity and improve memory,^71^ providing a link between our findings and previous animal research.

#### MEG recording

Participants were dressed in metal-free clothing and seated in a dimly lit, magnetically shielded recording booth. Visual stimuli were delivered via rear projection onto a semi-transparent screen using an DLP-LED-projector (ProPixx, VPixx Technologies Inc., Saint-Bruno, QC, Canada; resolution: 1920 × 1080) located outside the booth. The screen was positioned at a viewing distance of 100 cm from the participant. The display resolution was set to 1920 × 1080 pixels, with each letter subtending approximately 3 × 3.5 cm. Responses were collected using an MEG-compatible VPixx response system (VPixx Technologies Inc., Saint-Bruno, QC, Canada). MEG data were acquired using a whole-head Elekta Neuromag TRIUX system (Elekta Oy, Helsinki, Finland), equipped with 102 magnetometers and 204 planar gradiometers, while participants remained seated. Preparation and recordings took about 3 h.

#### Preprocessing and artifact rejection

Maxwell filtering was applied to suppress external noise, and the MEG data, originally sampled at 1000 Hz, were downsampled to 500 Hz. All analyses were conducted using MATLAB 2016b (Mathworks, Natick, USA). We included the 102 magnetometers in our analyses but no gradiometers. All filtering steps (details below) were performed with zero phaseshift IIR filters (fourth order butterworth filter; filtfilt.m in MATLAB). The data were first bandpass-filtered between 1 and 200 Hz. Cardiac and ocular artifacts were then removed using Independent Component Analysis (ICA) from the FieldTrip toolbox^72^ Subsequently, a notch filter was applied to eliminate line noise at 50 Hz and its second and third harmonics. To reject trials with excessive, non-physiological amplitude, we used an individual threshold for each subject. Mean variance across time and channels was calculated for each trial. Trials whose variance exceeded four standard deviations from the overall mean were excluded from further analysis. Finally, the continuous MEG data were segmented into epochs spanning from −2 s to 3 s relative to both cue and target onset.

#### Behavioral performance

Behavioral performance was assessed using DA, RTs and RT variance. DA and RTs capture complementary aspects of perceptual decision-making: DA primarily reflects sensory and decisional accuracy, whereas RTs are more sensitive to response dynamics and motor preparation. RT variance provides an index of trial-to-trial fluctuations in attentional stability and cognitive control. Examining all three measures allowed us to determine whether PA–related effects were expressed in perceptual accuracy, response speed, or both. We first tested whether cueing affected performance by comparing cued and uncued trials (see Figure 1B; Figure S1A), and whether PA improved performance for both cue types (see Figures S1B and S1C), using paired-sample two-tailed t-tests. We then compared overall behavioral performance between the PA and rest sessions across all trials using paired-sample two-tailed *t* test (see Figure 1C) and Bayes factor analysis. We adopted both *t* test and Bayes factor analysis because they address different aspects of the same comparison, and reporting them together provides a clearer and more balanced interpretation. The *t* test quantifies whether the observed difference would be unlikely under the null versus the alternative. This combination prevents common misinterpretations of non-significant *p*-values, allows readers to see whether the data actually support the null hypothesis or are simply inconclusive, and offers both a frequentist and an evidence-based perspective on the same effect.

#### Individual speed

The speed profile was recorded using the VPixx response system during MEG acquisition. It was calculated based on the light barrier onsets, covering the interval from 15 s before the first movement trigger to 15 s after the last movement trigger. Because triggers included both forward and downward movements, triggers with even indices were excluded to consider forward-and backward movement as one step. Raw speed traces were smoothed using a 5-s sliding window with 500-ms step increments. To assess walking-speed consistency across sessions (see Figure 1D), Pearson’s correlation coefficients of speed time series were computed between sessions. For visualization of mean speed profiles (see Figure S2A), the smoothed data were further processed with a 6-point moving average (500 Hz). The relationship between averaged speed and task performance was assessed using Spearman’s rank correlation for the PA session (see Figure 1E) and the rest session (see Figure S2B). Note that speed data are missing for two participants due to battery failure in the light-beam recording system.

#### Theta rhythmic modulations in behavioral performance

To examine behavioral performance across cue–target intervals (see Figure 2A), data were sorted by the cue-to-target interval length using a 50-ms window. This window length was chosen to match the temporal scale of theta-band dynamics (4–8 Hz; cycle durations ∼125–250 m s), providing sufficient temporal resolution to capture phase-dependent fluctuations while ensuring a sufficient number of trials per bin for reliable estimation. Shorter windows would reduce statistical reliability and increase the likelihood of empty bins, whereas longer windows would risk smoothing out theta-related temporal structure. The window was advanced in 2 m s steps across the 1000–2000 m s interval to densely sample the cue–target range and reconstruct a smooth, continuous behavioral modulation function over time.^6^ Because DA showed a ceiling in some conditions, we excluded participants with exceptionally stable performance to avoid ceiling-related distortions. Specifically, participants whose average DA variance across both sessions fell below the 20th percentile (*n* = 6) were removed from the cue-to-target delay analysis. The exclusion described above was applied only to the DA analyses; RTs and RT variance shown in Figure 2 were analyzed using the full dataset.

To derive the fitted cosine wavelet (see Figure 2A), we first detrended and z-scored the cue-to-target delay data. For visualization, the data were smoothed using a 10 point moving average (476 Hz). For cosine fitting, the data were further smoothed with a 20 point moving average to reduce noise. We performed data fitting using the MATLAB function fit.m, applying a cosine function of the form:

y=A·cos(2π·f·x+φ)+c,

In the fitting procedure, A was initially set to 3, c to the average of the data, and φ to the phase corresponding to the first identified valley in the data. f denotes the frequency of the cosine wavelet. To obtain the best-fitting result, f was varied from 4 to 8 Hz in 0.1 Hz steps. The fit was evaluated, and the one yielding the minimum mean squared error (MSE) was selected as the best fit.

To investigate frequency differences (see Figure 2B), behavioral data across different cue-to-target intervals were transformed into power spectra. The preprocessing steps included de-trending the data and subsequently smoothing it using a three-point moving window. PSD were then extracted using the Fast Fourier Transform (FFT). To attenuate the 1/f background activity, spectral estimates for each frequency of interest were multiplied by their respective center frequencies. The resulting spectra were further smoothed across neighboring frequency bins. For the DA data, *Z* score normalization was applied prior to de-trending. For statistical comparison (see Figure 2C), we averaged the PSD around the amplitude peak within theta range (5–7 Hz) and performed paired-sample one-tailed t-tests to compare PA and rest sessions, based on the *a priori* hypothesis that theta power would be greater in the PA session. Statistical significance was set at α = 0.05. For visualization, the spectra were interpolated to ten times the original resolution and smoothed using a five-point moving average. We computed PA spectra minus rest spectra for each subject. The resulting difference spectra were averaged across subjects and then z-scored within the 0–20 Hz range and results with and without participant exclusions are shown in Figure S4.

#### Temporal dynamics of PA-induced rhythmicity

To examine how rhythmicity evolved across the experimental session, we estimated the PSD using successive 2-min sliding windows spanning the interval from 1 to 7 min. We averaged the behavioral performance based on the cue-target interval for each minute, according to the blocks. The resulting data were then converted into PSD following the same procedure. For each participant and interval, PSD values were averaged within the 5–7 Hz frequency band. Temporal changes in rhythmicity were then quantified by fitting a linear regression to the interval-wise values, yielding an individual slope parameter. This analysis was performed separately for the rest and PA sessions (see Figure S3A, left). In a first step, we tested whether slope parameters for either condition differed significantly from zero using one-sample t-tests, conducted separately for DA and RTs. If a significant deviation from zero was observed in either session, slopes were compared between rest and PA using paired t-tests (see Figure S3A, right; Figure S3B).

#### Target response

To acquire the HFA amplitude data, we applied a Butterworth bandpass filter (80–150 Hz) to the epoched MEG signal and calculated the absolute value of its Hilbert-transformed signal. Baseline correction was performed from −100 m s to 0 m s before target onset for subsequent analyses. For the topography figure (see Figure 3A), we first z-scored the amplitude data and epoched it from 0 to 400 m s after stimulus onset. The data were averaged across all trials and sessions, revealing a strong HFA signal A_(t)_ signal in the occipital region. To examine potential differences in HFA amplitude between the PA and rest sessions, we selected three occipital sensors that showed robust HFA activity. Sensors were selected according to a threshold criterion, whereby z-scored HFA amplitude exceeded 1.5 across the sensor array. Importantly, the selected sensors formed a contiguous occipital cluster, ensuring that the analyses reflected a localized region of interest. For comparison of HFA amplitude between sessions (see Figure 3B), data were averaged across the three sensors from 100 to 300 m s after target onset, and a paired-sample *t* test was performed between the two sessions.

To investigate whether HFA amplitude reduced stimulus-unrelated noise following PA, we computed the temporal consistency of HFA amplitude around HFA peaks (200 m s) across different participants. We calculated Pearson correlations between all possible pairs of participants, yielding 465 inter-subject correlation values for both the PA and rest sessions. The correlations were computed for different epoched time windows around the peak, ranging from ±0.1 to ±0.2 s in 0.01-s steps. The resulting log_10_ *p*-values from paired-sample *t* test (comparing PA vs. rest across epochs) are shown in Figure S5 *(left)*. Individual R-values are plotted for the epoch range showing the strongest group difference (60–340 m s; see Figure S5, right).

#### Theta modulation after cue onset

To compare theta modulation across brain regions, we extracted theta amplitude from four regions of interest (frontal, temporal, parietal, and occipital; see Figure 4; Figure S6). Frontal and parietal cortices were selected based on prior evidence showing that rhythmic activity within the frontoparietal network supports rhythmic attentional sampling and that lesions of the prefrontal cortex impair theta-rhythmic attentional sampling in patients with chronic focal lesions.^6^

In addition, task-related HFA effects were strongest in occipital cortex in the present study, motivating the inclusion of occipital ROIs to directly relate theta dynamics in control networks to sensory processing. We opted for a small set of large ROIs spanning frontal, parietal, temporal, and occipital regions to enhance robustness and avoid overinterpretation.

Epoched MEG signals during the cue-target period were bandpass-filtered between 4 and 8 Hz using a Butterworth filter. The analytic signal was obtained via the Hilbert transform, and theta amplitude was defined as the absolute value of the resulting complex signal. Amplitudes were then averaged across sensors within each predefined brain region.

Session differences were computed as the individual PA-minus-rest contrasts and then averaged across participants (see Figure 4B, right). For statistical comparison, mean theta amplitudes were calculated for each participant between 360 and 1000 m s after cue onset and compared between PA and rest sessions using paired-sample t-tests. The 360–1000 m s post-cue interval was selected to isolate sustained theta-band activity following cue presentation. Early post-cue periods are dominated by cue-evoked sensory responses and phase-reset effects.^73^^,^^74^ We began our analysis at 360 m s to move beyond this transient phase and capture subsequent theta cycles. In our data, peak behavioral modulation occurred at ∼6 Hz (cycle duration ∼166 m s). Accordingly, the selected window spans at least two theta cycles (≈5.5–8 Hz), ensuring that the analyzed activity reflects sustained rhythmic dynamics rather than residual cue-locked responses.

To examine the relationship between frontal theta amplitude and the theta modulaiton of behavioral performance (see Figure 4C), the averaged theta amplitude across all frontal sensors (360–1000 m s) was correlated with theta power of RTs (4–8 Hz), obtained from power spectral analysis using Spearman’s rank correlation.

We quantified the modulation of HFA amplitude by ongoing theta phase during the cue–target interval (see Figure S7). Analyses were conducted on the theta phase of 24 frontal sensors and HFA amplitude of 3 occipital sensors of interest within the same cue-to-target interval. For each trial, the ongoing theta phase (4–8 Hz) was extracted from the frontal sensors using a Hilbert transform after band-pass filtering. Theta phase estimates were computed in the cue–target interval and sorted into 50 equally spaced phase bins (−π to π). For each theta phase bin, HFA amplitudes within the corresponding time frames were averaged across occipital sensors. Phase–amplitude coupling (PAC) was assessed by quantifying systematic modulation of HFA amplitude across theta phase bins. For each subject, we obtained 50-bin HFA modulation profiles for each pairing of 24 frontal and 3 occipital sensors, separately for both experimental conditions (rest and active movement). To identify frontal sensors showing the strongest theta-driven modulation of HFA, we collapsed HFA amplitude across the three occipital sensors and across subjects and conditions. This yielded a condition-independent grand-average HFA–theta phase relationship for each frontal sensor. A cosine function was fit to each grand-average modulation profile:

$$A\left(\phi \right)=a\cdot \cos\left(\phi -\phi_{0}\right)+c$$

where *A*(*ϕ*) is the HFA amplitude as a function of theta phase *ϕ*, and *a*, *ϕ*_0_, and *c*are free parameters. Model fit quality was quantified by root-mean-square error (RMSE), serving as an inverse measure of modulation strength. Based on a median split of RMSE values across the 24 frontal sensors, the 12 sensors with the smallest RMSE (i.e., strongest modulation) were selected for further analysis. For each subject and condition, HFA amplitude was averaged across the 12 selected frontal sensors, yielding subject-specific HFA distributions over 50 theta phase bins. For each subject and condition, the theta phase corresponding to the maximal HFA amplitude was identified as the preferred coupling phase. To compare the distribution of preferred coupling phases between conditions (rest vs. active movement), we assessed phase concentration parameter *κ* and circular mean phase direction. A Watson–Williams test, which was implemented in the Circular Statistics Toolbox, was applied to determine whether preferred phases differed significantly between conditions.

#### Theta rhythmic modulations of HFA

Next, we sorted the averaged HFA amplitude signals from 100 to 300 m s according to the cue-to-target interval, following the same approach used for the behavioral data (see Figure 5A). To obtain the HFA amplitude PSD, we first applied a Fast Fourier Transform (FFT) to the time-varying HFA amplitude signals. This transformation followed the same procedure described previously. We then averaged the resulting PSD within the 100–300 m s time window to obtain the mean PSD (see Figure 5B). Finally, PSD in the 5–7 Hz range were averaged and compared between sessions using a one-tailed paired-sample *t* test (see Figure 5C). To investigate the similarity of frequency results, we computed the differences in PSD of HFA amplitude and behavioral data between the PA and rest sessions (see Figure S4). PSD from the rest session were subtracted from those of the PA session, and the resulting differences were averaged and then z-scored across the 0–20 Hz range.

#### HFA fluctuations track behavioral oscillations under PA

To investigate the relationship between HFA amplitude and behavioral measures, we computed Pearson correlation coefficients between the cue-to-target interval sorted behavioral results and the corresponding HFA amplitude for each participant (see Figure 5D). The resulting R-values are plotted in Figure 5E.

### Quantification and statistical analysis

All statistical analyses were performed in MATLAB R2016b. Behavioral performance was quantified using DA, RTs, and RT variance. Group-level comparisons between cued and uncued trials (see Figures 1B and S1), and between PA and rest sessions (see Figures 1C, 3B, 4B, 4C, 5E, S5, and S6), were conducted using paired-sample two-tailed t-tests. Where an *a priori* directional hypothesis was specified (theta-band PSD comparisons), one-tailed paired-sample t-tests were used (see Figures 2C, 5C, and S3). All analyses were conducted at the participant level (*n* = 31), with the exception of DA rhythmicity analyses, from which six participants with DA variance below the 20th percentile were excluded to avoid ceiling-related distortions (*n* = 25 for that analysis). All t-tests were complemented by Bayes factor analysis (BF_10_) to distinguish between absence of evidence and evidence of absence for non-significant results. Additionally, effect sizes (η^2^), and 95% confidence intervals are reported.

The correlation between individual walking speed and behavioral performance was assessed using Spearman’s rank correlation (see Figures 1E and S2B). The correlation between frontal theta amplitude and the theta power of RTs was also assessed using Spearman’s rank correlation (see Figure 4C). Correlations between HFA amplitude fluctuations and behavioral oscillations, both derived as continuous time courses sorted by cue-to-target interval, were quantified using Pearson correlation coefficients (see Figure 5E). Inter-participant temporal consistency of HFA amplitude was assessed using Pearson correlation coefficients across all possible participant pairs (*n* = 465 pairs; see Figure S5). Preferred theta–HFA coupling phases were compared between PA and rest conditions using the Watson–Williams test implemented in the Circular Statistics Toolbox.
