# Supplementary material for: Physical activity enhances theta-periodicity of visual attentional allocation
Source: iScience. 2026 Jun 8;29(6):116240. doi: 10.1016/j.isci.2026.116240 (PMC13264037; doi:10.1016/j.isci.2026.116240)
Supplement: Document S1. Figures S1–S7 and Table S1 [file mmc1.pdf]

iScience, Volume *29*

Supplemental information

Physical activity enhances theta-periodicity of visual attentional allocation

Xinyun Che, Christoph Reichert, Robert T. Knight, and Stefan Dürschmid

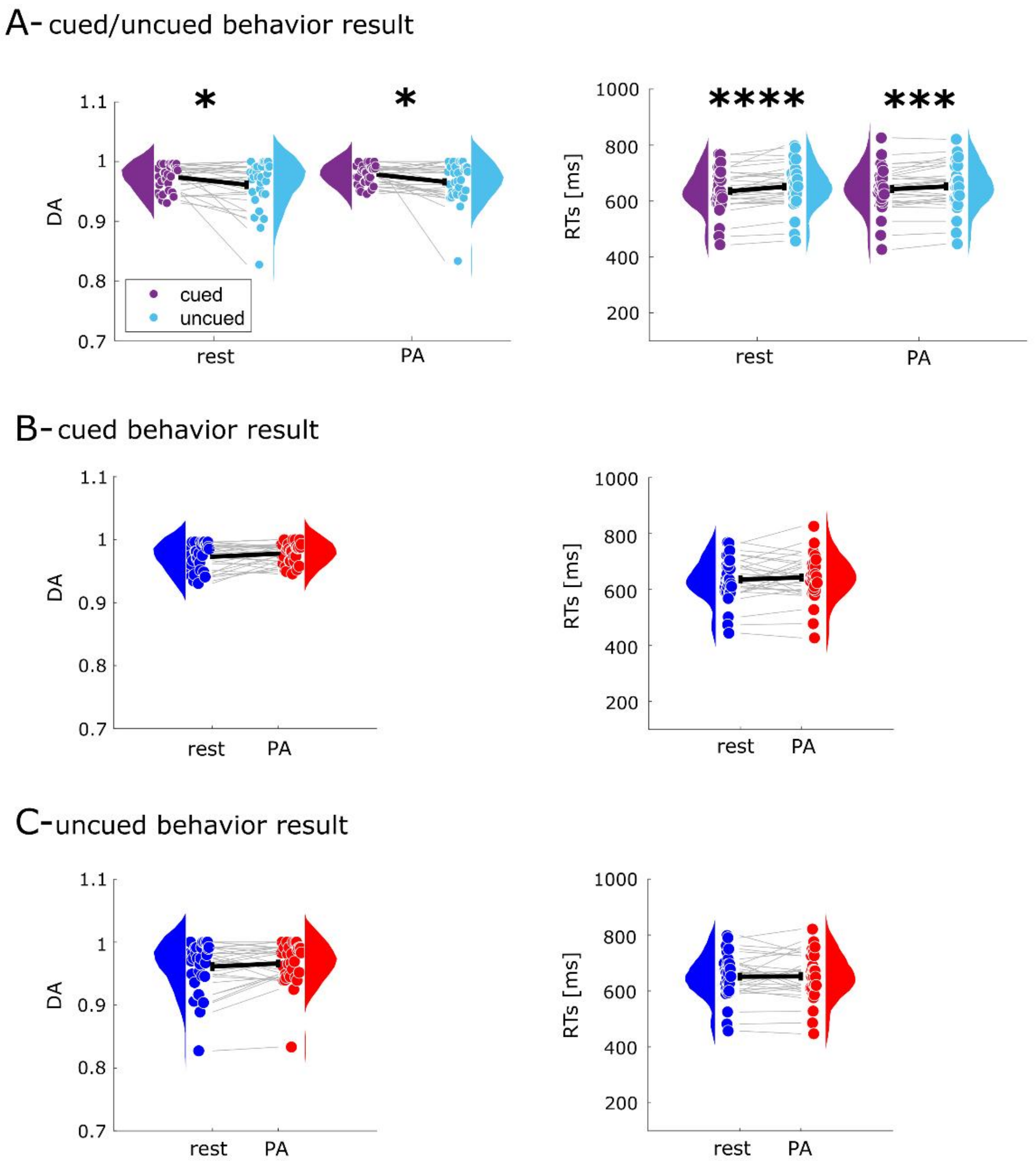

Figure S1. Behavioral results for cued and uncued trials, related to Figure 1B
A: Individual behavioral performance for cued and uncued trials, shown separately for rest and PA sessions (*p < 0.05, *** p<0.001, ****p < 0.0001). ***B***: Individual behavioral results from cued trials. No significant difference between PA and rest, either in DA (left: $t_{(30)} = 1.64$, $p = 0.11$, $BF_{10} = 0.64$) or RTs (right: $t_{(30)} = 0.85$, $p = 0.405$, $BF_{10} = 0.27$). ***C***: Individual behavioral results from uncued trials. No difference was found between PA and rest in uncued trials, either in DA (left: $t_{(30)} = 1.41$, p= 0.17, $BF_{10} = 0.47$) or RTs (right: $t_{(30)} = 0.13$, $p = 0.9$, $BF_{10} = 0.19$).

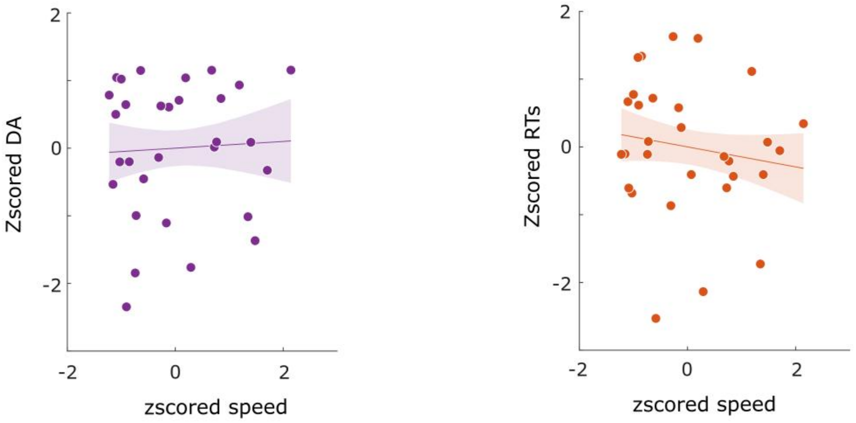

Figure S2. Correlation analysis between speed and behavioral performance from rest session, related to Figure 1E

(Left): Correlation coefficients between individual speed and DA. (Right): Correlation coefficients between speed and RTs.

A- DA power across successive minutes

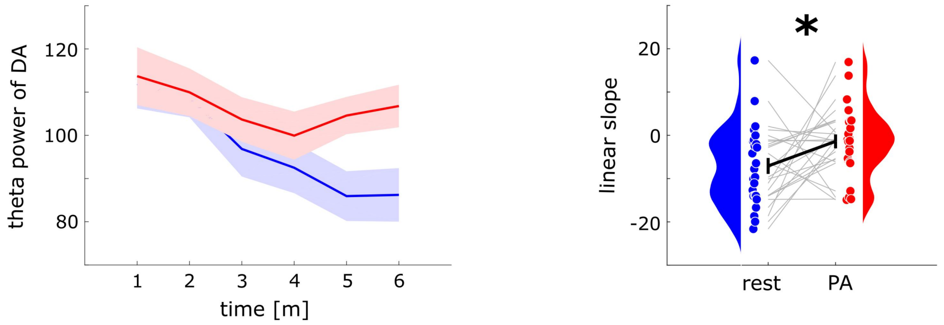

B-RTs power across successive minutes

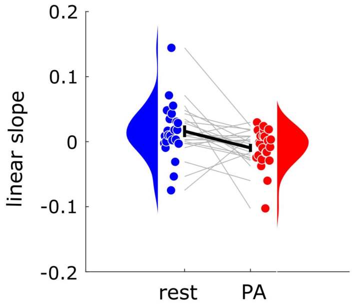

Figure S3. Frequency components from 5–7 Hz across different minutes, related to Figure 2
A (Left): The averaged 1/f-corrected power spectra of DA in the 5–7 Hz range are plotted across individual minutes. Red lines represent signals from PA session, while blue lines represent signals from rest session. Error bars indicate the standard error of the mean. (Right): Scatter plot of the linear slopes from the linear regression of DA power spectra across successive minutes for each individual subject (*$p < 0.05$). DA rhythmicity significantly decreased during rest ($t_{rest} = 3.8$, $p = 0.0008$), whereas no such effect was observed following PA ($t_{PA} = 0.87$, $p = 0.39$). Moreover, the change in DA rhythmicity differed significantly between rest and PA ($t = 2.15$, $p = 0.042$). ***B***: Scatter plot of the linear slopes from the linear regression of RTs power spectra across different minutes for each individual subject. For RTs, slope parameters did not differ from zero ($t_{rest} = 1.5$, $p = 0.12$; $t_{PA} = 0.29$, $p = 0.76$), indicating that PSD values showed no systematic trend over time.

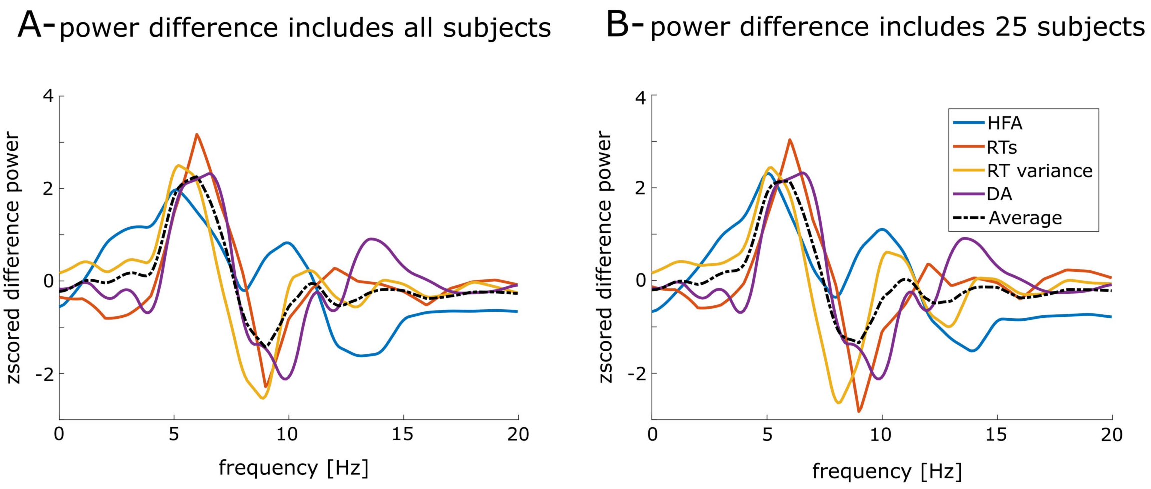

Figure S4. Spectral differences between PA and rest sessions (0-20 Hz), related to Figure 2B and Figure 5B

A: Z-scored PSD differences (all subjects; DA subset: n=25). ***B***: Z-scored PSD differences (n=25).

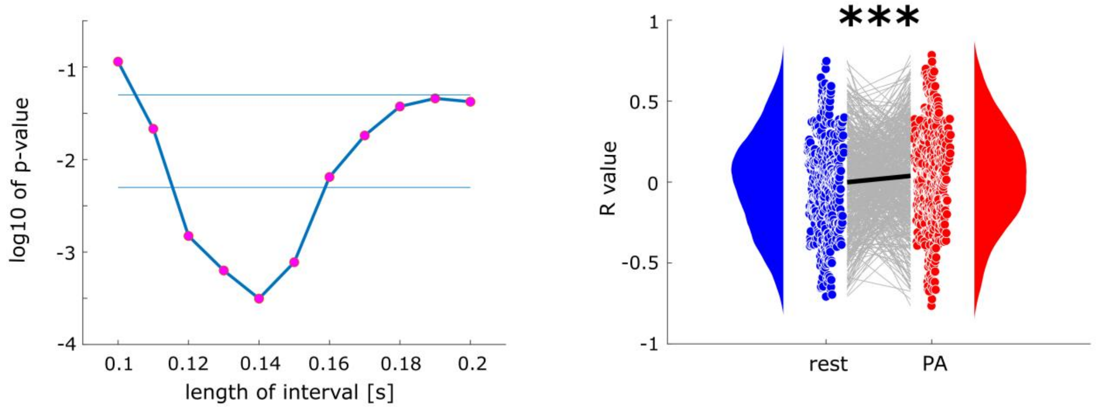

Figure S5. Correlation of HFA time courses across subjects, related to Figure 3B
(Left): $\log_{10}$ P-values from paired-sample t-test comparing PA vs. rest across epochs for different time windows centered around the HFA amplitude peak (200 ms). (Right): Inter-participant correlations of HFA amplitude within the 60–340 ms time window after target onset. Red represent signals from PA session, while blue represent signals from rest session (***p < 0.001).

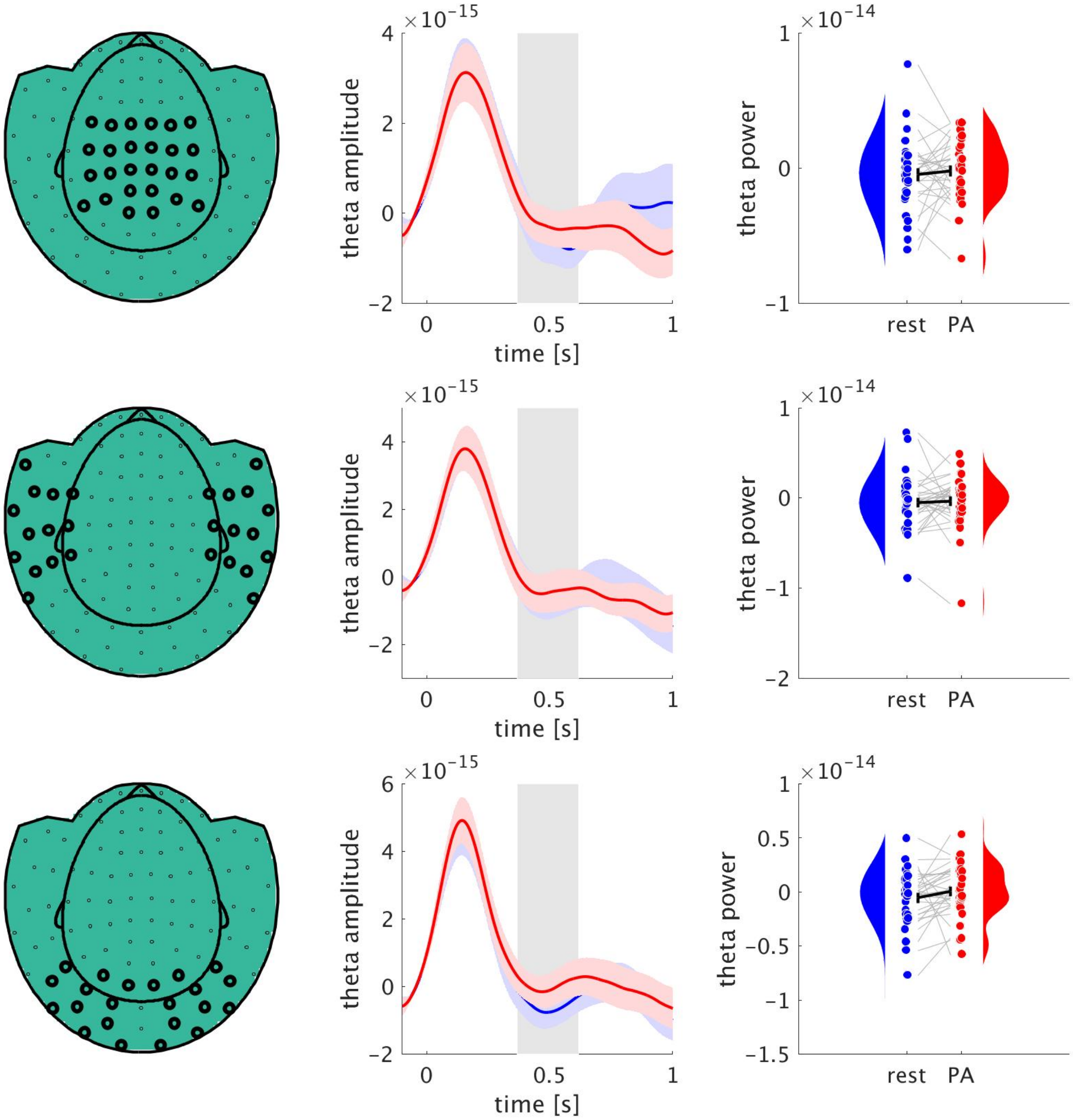

Figure S6. Theta amplitude across cortical regions following cue onset, related to Figure 4A
(Left): Sensor layout across cortical regions. (Middle): Grand-average theta amplitude time course aligned to cue onset. The shaded window indicates the time interval used for individual theta amplitude comparisons. Red and blue indicate PA and rest sessions, respectively. (Right): Mean theta amplitude for each participant, averaged across the shaded time window shown in the middle panel. Error bars represent the standard error of the mean.

Theta amplitude was compared between rest and PA across temporal, parietal, and occipital regions. No significant effects were observed in any of the three regions (*temporal*: $t_{(30)} = 0.569$, $p = 0.622$, $BF_{10} = 0.22$; *parietal*: $t_{(30)} = 0.24$, $p = 0.815$, $BF_{10} = 0.2$; *occipital*: $t_{(30)} = 1.32$, $p = 0.196$, $BF_{10} = 0.42$).

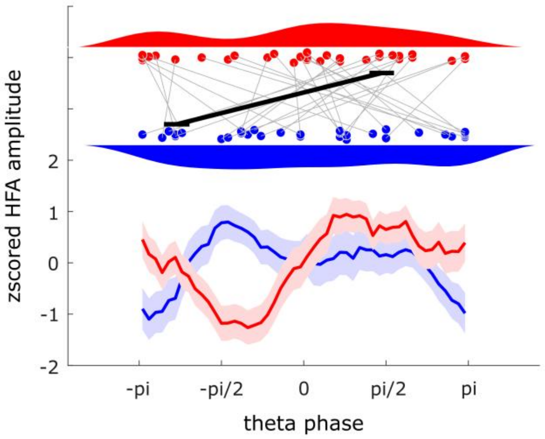

Figure S7. Frontal theta phase and occipital HFA amplitude coupling after cue onset

The averaged occipital HFA amplitude align with different frontal theta phase. The top scatter plot indicates individual dominant phases. Red and blue denote PA and rest sessions, respectively. Circular means were computed using circ_mean, and error bars represent the circular standard error of the mean.

Measure	Gender	rest	PA	Gender Effect	Interaction Effect
DA	female	0.96	0.97	F = 6.06,	F = 0.23,
	male	0.98	0.98	p = 0.02	p =0.64
RTs	female	645.37 ms	643 ms	F = 0.02,	F = 0.2,
	male	632.92 ms	649.33 ms	p = 0.88	p = 0.65
DA-theta	female	88.19	108.29	F = 0.06,	F = 0.03,
PSD	male	87.49	103.87	p = 0.8	p = 0.85
RT-theta	female	7.47×10^{4}	9.82×10^{4}	F = 0.22,	F = 0.15,
PSD	male	5.95×10^{4}	9.69×10^{4}	p = 0.64	p = 0.09
HFA-theta	female	1.74×10^{-25}	1.97×10^{-25}	F = 0.75,	F = 0.1,
PSD	male	1.55×10^{-25}	1.89×10^{-25}	p = 0.39	p = 0.76

Table S1. Gender differences in behavioral performance and averaged theta power following PSD analysis

The table summarizes the mean behavioral performance and theta power for female and male participants. Gender and Gender × movement (*PA* vs. *rest*) interaction effects were assessed using two-way ANOVAs.

The sample included 19 female and 12 male participants. To assess potential gender effects, we conducted two-way ANOVAs with gender and movement condition (*PA* vs. *rest*) as factors on behavioral performance (*RTs* and *DA*).

For RTs, no significant main effect of gender (*female*: 644.18 ms, *male*: 641.13 ms) and no gender × movement interaction were observed. For DA, a significant main effect of gender was found (F = 6.06, p = 0.02), whereas no significant gender × movement interaction was observed (F = 0.23, p = 0.64). Mean DA values were 0.96 (rest) and 0.97 (PA) for females, and 0.98 (rest) and 0.98 (PA) for males. However, the primary behavioral outcomes and neural-behavioral relationships reported in this study were driven by RT measures; therefore, the observed gender difference in DA does not affect the main conclusions.

Furthermore, no significant main effects or interactions involving gender were observed in averaged theta PSD for DA (*female*: PA: 108.29, rest: 88.19; *male*: PA: 103.87, rest: 87.49; gender: F = 0.06, p = 0.80; interaction: F = 0.03, p = 0.85), RTs (*female*: PA: 9.82×10^{4}, rest: 7.47×10^{4}; *male*: PA: 9.69×10^{4}, rest: 5.95×10^{4}; gender: F = 0.22, p = 0.64; interaction: F = 0.15, p = 0.09), or HFA (*female*: PA: 1.97×10^{-25}, rest: 1.74×10^{-25}; *male*: PA: 1.89×10^{-25}, rest: 1.55×10^{-25}; gender: F = 0.75, p = 0.39; interaction: F = 0.10, p = 0.76). Together, these results indicate that gender did not influence the behavioral or neural effects reported in this study.
